# Age added to MELD or ACLF predicts survival in patients with alcohol-associated hepatitis declined for liver transplantation

**DOI:** 10.1097/HC9.0000000000000514

**Published:** 2024-08-19

**Authors:** Stephanie M. Rutledge, Rohit Nathani, Brooke E. Wyatt, Erin Eschbach, Parth Trivedi, Stanley Kerznerman, Lily Chu, Thomas D. Schiano, Leona Kim-Schluger, Sander Florman, Gene Y. Im

**Affiliations:** 1 Department of Medicine, Division of Liver Diseases, Recanati/Miller Transplantation Institute, Icahn School of Medicine at Mount Sinai, New York, New York, USA; 2 Department of Medicine, Mount Sinai Morningside and West, New York, New York, USA; 3 Department of Medicine, Division of Liver Diseases, Icahn School of Medicine at Mount Sinai, New York, New York, USA; 4 Department of Medicine, Icahn School of Medicine at Mount Sinai, New York, New York, USA; 5 Department of Surgery, Division of Abdominal Transplantation, Recanati/Miller Transplantation Institute, Icahn School of Medicine at Mount Sinai, New York, New York, USA

## Abstract

**Background:**

Severe alcohol-associated hepatitis (AH) that is nonresponsive to corticosteroids is associated with high mortality, particularly with concomitant acute-on-chronic liver failure (ACLF). Most patients will not be candidates for liver transplantation (LT) and their outcomes are largely unknown. Our aim was to determine the outcomes of these declined candidates and to derive practical prediction models for transplant-free survival applicable at the time of the waitlist decision.

**Methods:**

We analyzed a database of patients with severe AH who were hospitalized at a LT center from January 2012 to July 2021, using the National Death Index for those lacking follow-up. Clinical variables were analyzed based on the endpoints of mortality at 30, 60, 90, and 180 days. Logistic and Cox regression analyses were used for model derivation.

**Results:**

Over 9.5 years, 206 patients with severe AH were declined for LT, mostly for unfavorable psychosocial profiles, with a mean MELD of 33 (±8), and 61% with ACLF. Over a median follow-up of 521 (17.5–1368) days, 58% (119/206) died at a median of 21 (9–124) days. Of 32 variables, only age added prognostic value to MELD and ACLF grade. CLIF-C ACLF score and 2 new models, MELD-Age and ACLF-Age, had similar predictability (AUROC: 0.73, 0.73, 0.72, respectively), outperforming Lille and Maddrey’s (AUROC: 0.63, 0.62). In internal cross-validation, the average AUROC was 0.74. ACLF grade ≥2, MELD score >35, and age >45 years were useful cutoffs for predicting increased 90-day mortality from waitlist decision. Only two patients initially declined for LT for AH subsequently underwent LT (1%).

**Conclusions:**

Patients with severe AH declined for LT have high short-term mortality and rare rates of subsequent LT. Age added to MELD or ACLF grade enhances survival prediction at the time of waitlist decision in patients with severe AH declined for LT.

## INTRODUCTION

Alcohol-associated hepatitis (AH) is now the fastest rising indication for liver transplantation (LT) in the United States.^1^ Severe AH without response to medical therapy is a form of life-threatening alcohol-associated liver disease (ALD) with high short-term mortality rates, particularly in the presence of acute-on-chronic liver failure (ACLF).^2^,^3^,^4^ Alcohol-precipitated ACLF is a more severe subtype than other ACLF subtypes^5^ and is associated with lower 30-day transplant-free survival (22% vs. 53%).^6^ In patients with severe AH and ACLF (AH-ACLF), ACLF grade is associated with increased mortality and reduced likelihood of response to corticosteroids.^7^

While only a minority of severe AH nonresponders are eligible for LT as rescue therapy, the outcomes of the declined majority are largely unknown.^8^,^9^ This group often has multiorgan failure, making them ineligible for AH clinical trials and, thus, historically poorly characterized. A recent study demonstrated that younger age, lower peak Model for End-Stage Liver Disease (MELD) score, and lower index international normalized ratio were associated with spontaneous recovery in declined candidates with severe AH but lacked prognostic modeling and primary analyses regarding ACLF.^10^ Also, MELD-Na may also overestimate mortality risk compared to other indications for LT.^1^

As patients with severe AH declined for LT may survive to become acceptable candidates in the future or improve enough clinically to obviate LT, a deeper understanding of their outcomes is needed to avoid unnecessary LT in the current landscape of organ shortages. Our aim was to determine the outcomes of patients with severe AH declined for LT and to derive practical prediction models of transplant-free survival applicable at the time of waitlist decision.

## METHODS

### Study population

We identified patients over 18 years old with severe AH nonresponsive to medical therapy hospitalized at our center from January 2012 through July 2021 per protocol, as described.^9^,^11^ According to center protocol, all patients with severe AH not responding to or ineligible for medical therapies such as corticosteroids were discussed at our transplant selection meetings and recorded. Patients were included if they had probable or definite AH, as defined by the National Institute on Alcohol Abuse and Alcoholism (NIAAA) consensus criteria and MELD score >20, consistent with severe AH.^8^,^12^ While patients declined for LT for AH composed the primary cohort of analysis (n = 206), an additional cohort accepted for LT for AH but who died before LT was included for model derivation (n = 28), as their outcomes reflect the transplant-free natural history of severe AH.

### Patient outcomes and data

Patient electronic medical records (EMR; Epic Systems) were retrospectively reviewed. To determine postdischarge outcomes for those lacking follow-up in our EMR, we used the National Death Index (NDI), a centralized database of all deaths in the United States maintained by the Centers for Disease Control and Prevention. Data at the time of the waitlist decision were used to test and derive prediction models to provide a practical time point for analysis. We determined ACLF grade, Chronic Liver Failure-Consortium Organ Failure score (CLIF-OF), and CLIF Consortium ACLF score (CLIF-C ACLF) using the European Association for the Study of the Liver (EASL)-CLIF Consortium online calculators.^5^,^13^ CLIF-OF designates a subscore in the range of 1–3 for each organ system, with a final score ranging from 6 to 18. CLIF-C ACLF incorporates CLIF-OF plus age and white blood cell count. This study was approved by the Institutional Review Board of Mount Sinai. All research was conducted in accordance with both the Declarations of Helsinki and Istanbul.

### Statistical analysis and model derivation

Descriptive statistics, including mean, median, SD, and IQR, were used to determine the characteristics of the study cohort. Patient groups were compared using the chi-square test for categorical variables and the Student *t* test for continuous data. Kaplan-Meier (KM) survival analyses were used to assess mortality at 30, 60, 90, and 180 days from waitlist decision and to inform potential model cutoff points for age, MELD, and ACLF grade. Patients living beyond 180 days were censored at last follow-up or death.

Potential covariates significant at the bivariate level were then entered into a multivariable logistic regression model using backward elimination to establish a new model for mortality. To maximize practicality, we excluded any single variables common to MELD or ACLF scores to enhance the predictive ability of these existing models rather than creating a new model. Based on the baseline model, other clinically relevant adjusted models were tested for performance and compared with validated AH models, such as Maddrey’s discriminant function (DF) and Lille. Discrimination of each model was assessed using AUROC curves and compared to both ACLF models, baseline and derived, using the DeLong method.^14^ Internal cross-validation of each model was performed by randomly splitting the sample into 2 subsets, running each model, and calculating the relevant c-statistic. After establishing the new predictive models, Cox regression analyses were used to assess adjusted hazard ratios for mortality at 30, 60, 90, and 180 days after the waitlist decision.^15^

All tests were 2-tailed, and a *p* value of <0.05 was considered statistically significant. Statistical analysis was performed using R (2021, R Core Team, R Foundation for Statistical Computing; https://www.R-project.org/) and SPSS Statistics (version 28.0. IBM Corp). TRIPOD reporting guidelines were followed (Supplemental Data, http://links.lww.com/HC9/B14).^16^

## RESULTS

### Patient characteristics

**FIGURE 1:**
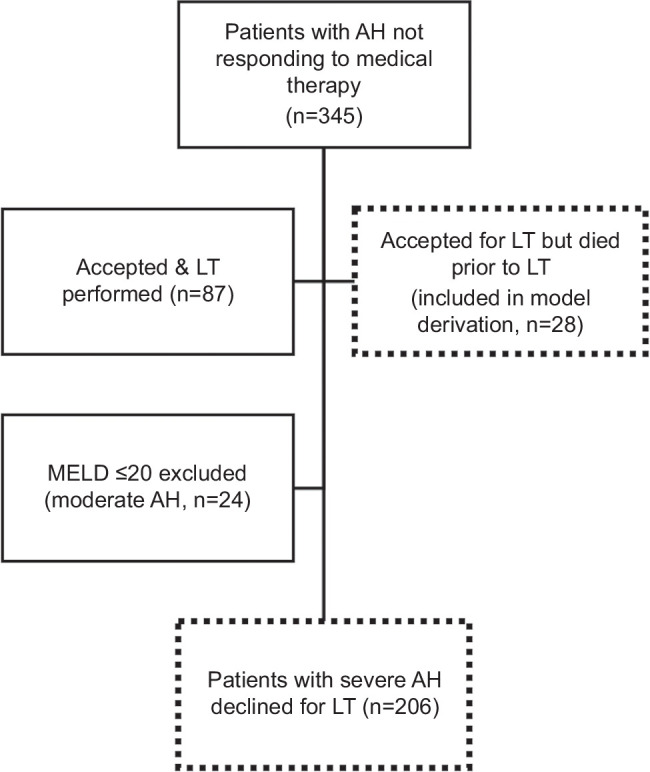
Study flow diagram. Abbreviations: AH, alcohol-associated hepatitis; LT, liver transplantation; MELD, Model for End-Stage Liver Disease.

Over 9.5 years, 345 consecutive patients with severe AH nonresponsive to medical therapy were presented at our transplant selection meeting: 87 underwent LT and 206 with MELD >20 were declined for LT (Figure 1). The most common reasons for LT decline were unfavorable psychosocial profiles (63%, 130/206) and clinical stability (committee consensus that the patient would recover without LT; 18%, 37/206). Of these, 15/37 were responders to medical therapy, according to the Lille score. The declined cohort had a mean age of 45.5 years (SD: ±11.1) with slight male and White predominance (56% and 54%, respectively). At the time of the waitlist decision, the cohort had mean scores of DF 114 (±63), MELD 33 (±8), Lille 0.54 (±0.33), and CLIF-C ACLF 50 (±10) (Table 1). For those who were not treated with corticosteroids, the Lille model was calculated using day 0 as the day of initial presentation. Most patients met the EASL-CLIF criteria for ACLF (61%, 126/206), with 71% (89/126) having an ACLF grade of 2 or 3. The median time from admission or transfer to our center to LT waitlist decision was 5 days (IQR: 3–10.5).

**TABLE 1:** Characteristics of patients with alcohol-associated hepatitis declined for liver transplantation

Variable	Mean (SD) or n (%)
Age (y)	45.5 (11.1)
Age ≥45 y	67 (32.5)
Sex (male)	116 (56.3)
Race/ethnicity	
White	111 (53.9)
Black	17 (8.3)
Hispanic/Latino	55 (26.7)
Asian	12 (5.8)
Other	4 (1.9)
Unknown	5 (2.4)
Death	119 (57.8)
Received corticosteroids	83 (40.3)
Reason for decline for liver transplantation	
Psychosocial	130 (63.1)
Medical contraindications	34 (16.5)
Clinical stability	37 (18.0)
Financial/insurance	3 (1.5)
Unknown	2 (0.9)
Presence of ACLF	126 (61.2)
ACLF grade	
0	80 (38.8)
1	37 (18.0)
2	53 (25.7)
3	36 (17.5)
MELD	32.5 (7.7)
MELD-Na	33.2 (7.0)
CLIF-C ACLF	50.1 (10.1)
DF	114.2 (62.5)
CLIF-OF	10.7 (2.3)
Lille score	0.54 (0.33)
Lille >0.45	130 (55.6)
Prothrombin time (s)	33.4 (13.1)
INR	2.5 (1.0)
WBC (×10^9^/L)	16.5 (10.3)
Total bilirubin (mg/dL)	23.3 (11.9)
Creatinine (mg/dL)	2.0 (1.4)
Renal replacement therapy	46 (22.3)
Sodium (mEq/L)	135 (4)
Albumin (g/dL)	2.8 (0.8)
MAP (mm Hg)	80.2 (11.5)
SpO_2_ (%)	97 (2)
FiO_2_	0.3 (0.1)
SpO_2_/FiO_2_	433.8 (82.4)
Total bilirubin categorized	
≤15	59 (28.6)
15.1–20	32 (15.5)
20.1–30	61 (29.6)
≥30.1	54 (26.2)
Creatinine categorized	
<1.5	107 (52.7)
1.5–1.9	19 (9.4)
≥2	77 (37.9)
MELD categorized	
≤30	91 (44.2)
30.1–35	37 (18.0)
35.1–40	44 (21.4)
≥40.1	34 (16.5)

*Note*: Clinical characteristics of patients with severe alcohol-associated hepatitis declined for liver transplantation at the time of waitlist decision (n = 206).

Abbreviations: ACLF, acute-on-chronic liver failure; CLIF-C ACLF, Chronic Liver Failure-Consortium Acute-on-Chronic Liver Failure score; CLIF-OF, Chronic Liver Failure-Consortium Organ Failure score; DF, Maddrey’s discriminant function; FiO_2_, fraction of inspired oxygen; INR, international normalized ratio; MAP, mean arterial pressure; MELD, Model for End-Stage Liver Disease; Na, sodium; SpO_2_, oxygen saturation; WBC, white blood cell count.

### Outcomes of the declined cohort

Follow-up data were captured in our EMR for half of the declined cohort (103/206), with the remaining half ascertained using the NDI. ALD was the cause of death in 81% (51/63) of the patients who survived to discharge: 12/14 determined from the EMR and 39/49 from the NDI. Other causes of death included HCC, cholangiocarcinoma, sepsis, and peritonitis. Over a median follow-up of 521 (17.5–1368) days, 58% (119/206) died, with 27% (56/206) of patients dying during the index admission. The median length of stay was 8.5 days (4–18). The cumulative incidence of death at 180 days was 45% (93/206) and corresponded with ACLF grade: ACLF-3, 81% (29/36); ACLF-2, 49% (26/53); ACLF-1, 32% (12/37); and ACLF-0, 33% (26/80). The median survival was 10 days (4–61) with ACLF-3 compared to 59 days (13–1052) with ACLF-2, 716 days (46–1641) with ACLF-1, and 692 days (71–1657) with ACLF-0. The log-rank test confirmed statistically significant differences in cumulative incidence of death, χ^2^(3) = 39.79, *p* < 0.001. These results prompted a closer look at death before the 180-day time point to investigate the most appropriate timeline for prognostication.

### Other postdischarge outcomes

Among surviving patients with available EMR data, 39% (18/46) had alcohol relapse (per NIAAA definitions) at a median of 12 months (0.2–55.5). Of the patients with available alcohol data who died after discharge, 11/12 (92%) were abstinent at the time of their terminal admission. The abstinent population died of a variety of causes, but mostly related to their underlying ALD: 5/11 had a gastrointestinal bleed, 4/11 had spontaneous bacterial peritonitis, and 6/11 had an acute kidney injury which was felt to be hepatorenal syndrome in 2/6. Two others had hepatic hydrothorax. We analyzed the predictors of relapse and found that a higher MELD was associated with reduced odds of relapse (OR: 0.97 [95% CI: 0.94, 0.99], *p* = 0.01). Other variables, including age (*p* = 0.97), sex (*p* = 0.82), race (*p* = 0.07), and ACLF grade at decline (*p* = 0.87) were not significant. Of those declined for psychosocial reasons with available alcohol data, 12/28 (43%) had relapsed. Relapse was not associated with mortality (*p* = 0.72).

Among the patients declined for clinical stability, 51% (19/37) were “fast fallers” (bilirubin improvement of >20% at 7 days).^17^ Seven out of 37 (19%) declined for clinical stability died after a median of 81 days (27–182); 6 were “fast fallers.” Reason for death was available for 6/7 and was ALD in 4/6, “intracranial abscess and granuloma” in 1, and sepsis in 1. We analyzed all “fast fallers” in the declined cohort, 73/206 (35%), and found that 36/73 (49%) died after a median of 21 days (10–102). “Fast fallers” had a trend toward lower mortality that did not reach statistical significance (−0.13 [0.07], *p* = 0.07). Trajectories of bilirubin (“fast faller,” “static,” and “rapid riser”) at the time of the waitlist decision were not associated with survival (*p* = 0.47).

Two (1%) patients initially declined for LT eventually underwent simultaneous liver-kidney transplantation after 125 and 350 days, respectively. The first was declined for frailty but waitlisted after improvement with physical rehabilitation. The second was declined for an unfavorable psychosocial profile but was waitlisted after completion of an alcohol rehabilitation program and 6 months of abstinence with biomarker testing. Both patients were abstinent and alive at the last follow-up of 433 and 738 days, respectively.

### Model derivation for mortality prediction in severe AH

**FIGURE 2:**
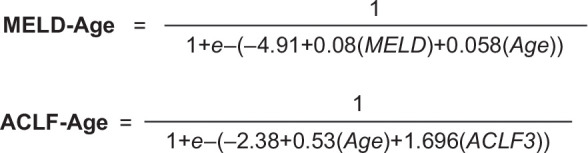
Formulas for MELD-Age and ACLF-Age models. Abbreviations: ACLF, acute-on-chronic liver failure; MELD, Model for End-Stage Liver Disease.

Using univariable analysis, we tested the prognostic values of 32 clinical and laboratory variables at the time of the waitlist decision for mortality (Supplemental Table S1, http://links.lww.com/HC9/B13). Twenty-two variables reached a *p* value <0.05 in univariable analysis (Table 2). Through backward elimination multivariable logistic regression analysis, age was found to enhance the predictive ability of the well-established models of MELD and ACLF grade (Supplemental Table S2, http://links.lww.com/HC9/B13). MELD itself was not significant in the presence of the CLIF-C ACLF score. Consequently, the newly proposed models were MELD-Age and ACLF-Age. Other adjusted models were tested for performance and compared with validated AH models. Using AUROC analysis, CLIF-C ACLF and the 2 new enhanced models, MELD-Age and ACLF Grade-Age (ACLF-Age), demonstrated similar predictability (AUROC: 0.73, 0.73, 0.72, respectively), which were significantly higher than those of the DF and Lille models (AUROC: 0.62, 0.63, *p* ≤ 0.04) (Supplemental Table S3, http://links.lww.com/HC9/B13). The formulas for MELD-Age and ACLF-Age are presented in Figure 2 and are available online at https://www.alcoholguidelines.org/calculators. Although the Lille model had high predictability (OR = 3.73, *p* < 0.001), its lower AUROC indicated good identification of true negatives but suboptimal performance with positive cases. Finally, we performed a sensitivity analysis to exclude patients with ventilation or vasopressor requirements (critical care variables of poor prognosis) and evaluated the remaining 4 organ subcomponents of CLIF-C ACLF. Liver and brain scores had the most significant impact on survival (Supplemental Table S4, http://links.lww.com/HC9/B13).

**TABLE 2:** Univariable logistic regression analysis of variables at waitlist decision for mortality

Variable	OR	95% CI	*p*
Age	1.06	1.03–1.08	<0.001
INR	1.05	1.03–1.08	<0.001
Creatinine (mg/dL)	1.45	1.17–1.79	0.001
Need for RRT	1.95	1.003–3.77	0.049
Albumin (g/dL)	1.57	1.10–2.24	0.01
SpO_2_/FiO_2_	0.99	0.98–1.00	0.03
MELD	1.09	1.05–1.13	<0.001
MELD-Na	1.09	1.05–1.14	<0.001
Maddrey’s DF	1.01	1.00–1.02	0.04
CLIF-C ACLF	1.11	1.06–1.15	<0.001
CLIF-OF	1.46	1.24–1.71	<0.001
Lille Score	3.74	1.62–8.62	0.002
West Haven Grade for HE			
0			0.07
1			0.05
2			0.05
3			0.09
4			0.99
Use of vasopressors	8.38	1.08 – 64.95	0.04
Mechanical ventilation	8.46	1.09–65.55	0.04
ACLF grade			
0			0.002
1	1.33	0.64–2.80	0.446
2	1.95	0.99–3.86	0.06
3	5.43	2.19–13.46	<0.001
Any ACLF criteria met	2.24	1.30–3.87	0.004
Liver score			
1			0.02
2			0.09
3	13.38	1.61–111.01	0.02
Liver dysfunction	2.68	1.30–5.52	0.01
Kidney score			
1			0.01
2	2.72	1.09–6.78	0.03
3	2.35	1.25–4.45	0.01
Kidney dysfunction	2.01	1.08–3.75	0.03
Brain score			
1			0.002
2	1.97	1.09–3.58	0.03
3	9.78	2.21–43.22	0.003
Brain dysfunction	7.78	1.78–33.95	0.01
Coagulation score			
1			0.08
2			0.15
3	2.04	1.08–3.86	0.03
Coagulation dysfunction			0.09
Circulatory score			
1			0.01
2	2.81	1.09–7.25	0.03
3	12.26	1.59–94.36	0.02
Circulatory dysfunction	10.92	1.42–83.81	0.02
Lung score			
1			0.05
2			1.00
3	12.67	1.66–96.68	0.01
Lung dysfunction	12.47	1.64–95.15	0.02
Total organ dysfunction			0.02
1			0.45
2			0.07
3	9.00	2.05–39.50	0.004
4			1.00
5	10.50	1.02–108.58	0.049
6			1.00

*Not*e: Univariable logistic regression analysis demonstrating the prognostic significance of 22 variables at the time of waitlist decision for mortality.

Abbreviations: ACLF, acute-on-chronic liver failure; CLIF-C ACLF, Chronic Liver Failure-Consortium Acute-on-Chronic Liver Failure score; CLIF-OF, Chronic Liver Failure-Consortium Organ Failure score; FiO_2_, fraction of inspired oxygen; INR, international normalized ratio; Maddrey’s DF, Maddrey’s discriminant function; MELD, Model for End-Stage Liver Disease; Na, sodium; RRT, renal replacement therapy; SpO_2_, oxygen saturation.

### Assessing practical thresholds for clinical use

**FIGURE 3:**
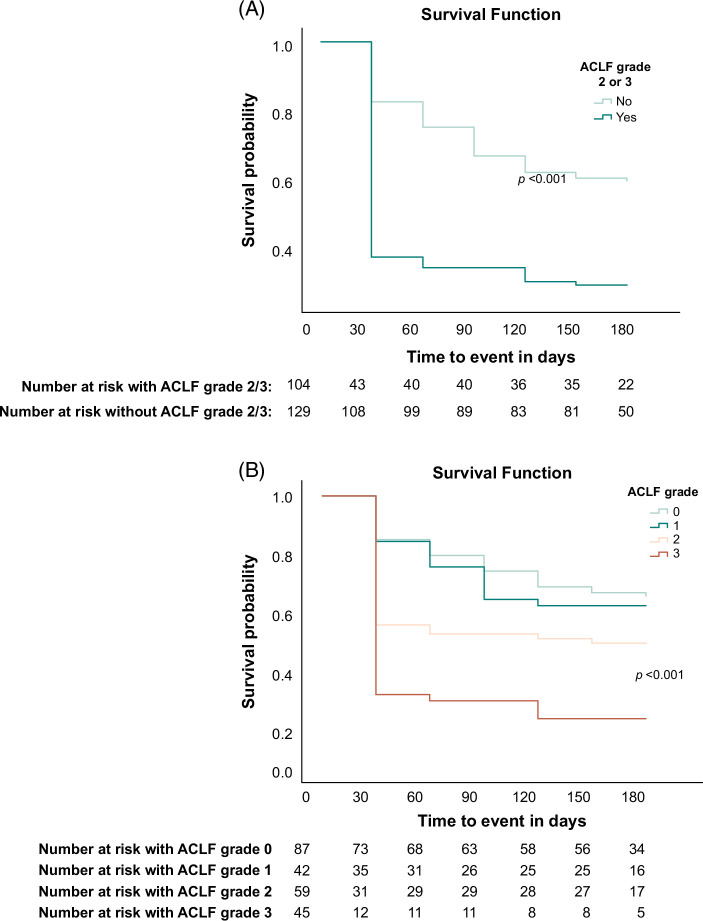
(A, B) Kaplan-Meier survival analysis according to ACLF grade 2 cutoff at the time of waitlist decision. Abbreviation: ACLF, acute-on-chronic liver failure.

**FIGURE 4:**
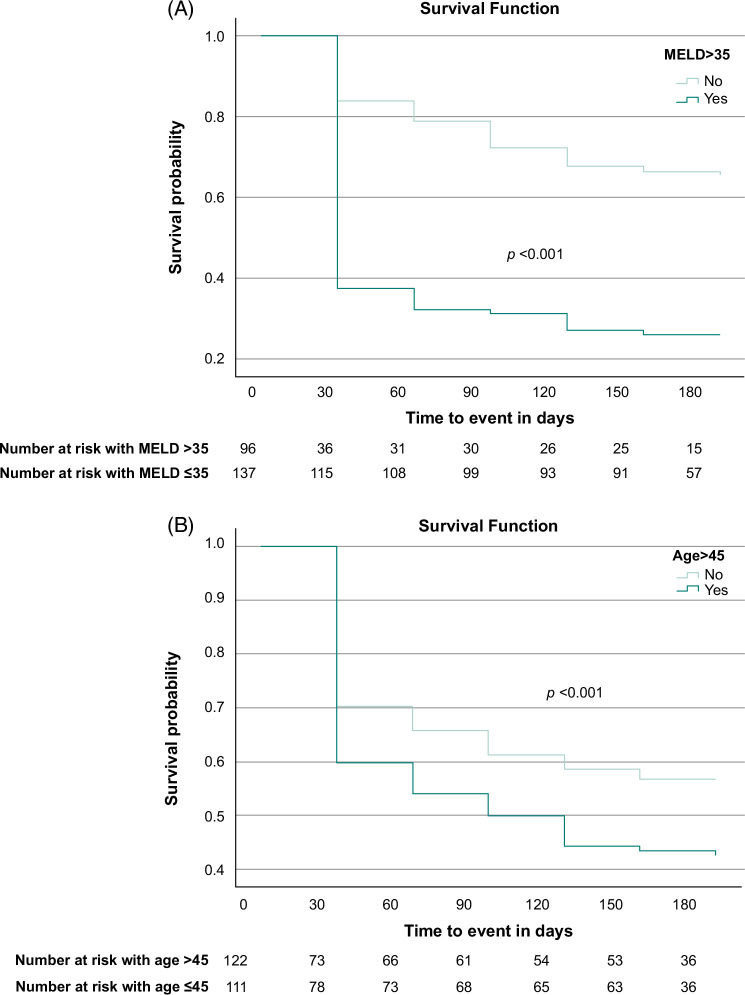
(A, B) Kaplan-Meier survival analysis according to MELD 35 and age 45 cutoffs at the time of waitlist decision. Abbreviation: MELD, Model for End-Stage Liver Disease.

As calculating the CLIF-C ACLF score can be complicated, we assessed ACLF grade, MELD, and age (significant covariates from the logistic regression models) individually to discriminate clinically useful cutoffs (Figures 3 and 4, Supplemental Table S5, http://links.lww.com/HC9/B13). KM survival analysis showed that patients with ACLF grades 0 and 1 had higher cumulative survival rates within the first 90 days after the waitlist decision. Pairwise comparisons showed significant differences in survival. Specifically, grades 2 and 3 differed significantly from all grades, whereas grades 0 and 1 were statistically insignificant. We then compared ACLF grade 2 or 3 with all other grades. Although KM analysis showed significant differences in cumulative survival of patients with ACLF grade ≥2 compared to ≤1 and was confirmed by regression analysis (OR = 2.6, 95% CI: 1.49–4.54, *p* < 0.001), it decreased overall predictability when entered back into the new model (AUROC = 0.70), although it was still higher than ACLF grade alone (AUROC = 0.64). This was also the case for the cutoffs investigated for MELD and age. Patients with MELD >35 had a 3.5-fold increased mortality compared to those with MELD ≤35 (95% CI: 1.93–6.25, *p* < 0.001). Patients aged >45 years had a 2.7-fold increase in mortality compared to those aged ≤45 years (95% CI: 1.56–4.65, *p* < 0.001). Despite their significance, they did not improve the overall model and were therefore not included.

### Internal validation

The ACLF-Age and MELD-Age models were validated using 2 randomly generated subsets of patients. Within subset 1 (n = 127), ACLF-Age correctly classified 69% of cases with an AUROC of 0.73, and MELD-Age correctly classified 67% of cases with an AUROC of 0.71. Within subset 2 (n = 120), ACLF-Age correctly classified 70% of cases with an AUROC of 0.76, and MELD-Age correctly classified 59% of cases with an AUROC of 0.75. These performance measures suggest that the 2 proposed models may adequately predict transplant-free survival but require external validation in an independent cohort.

### Assessing model time points

Considering the results of the KM survival analysis for both MELD and ACLF, Cox regression analyses were performed at 30, 60, 90, and 180 days after the waitlist decision to assess the most clinically relevant time point using the newly derived models (Table 3). For both MELD-Age and ACLF-Age, the greatest difference in the hazard of mortality was observed at the same or similar rates within the first 90 days after the waitlist decision. After controlling for age, those with ACLF-3 had 8.4 times increased odds of death within 30 days and 6.6 times within 60 days when compared to ACLF-0.

**TABLE 3:** Model performances of MELD-Age and ACLF-Age to predict survival at 30, 60, 90, and 180 days

	Within 30 days				Within 60 days				Within 90 days				Within 180 days			
	aHR	95% CI		*p*	aHR	95% CI		*p*	aHR	95% CI		*p*	aHR	95% CI		*p*
Model 1																
Age	1.03	1.01	1.05	**0.003**	1.03	1.01	1.05	**0.004**	1.03	1.01	1.05	**0.001**	1.03	1.01	1.05	**<0.001**
MELD	1.11	1.09	1.14	**<0.001**	1.11	1.08	1.13	**<0.001**	1.09	1.07	1.12	**<0.001**	1.09	1.07	1.12	**<0.001**
Model 2																
Age	1.03	1.00	1.05	**0.017**	1.02	1.00	1.04	**0.023**	1.03	1.01	1.04	**0.009**	1.03	1.01	1.04	**0.006**
ACLF grade																
0				**<0.001**				**<0.001**				**<0.001**				**<0.001**
1	1.09	0.44	2.69	0.858	1.25	0.60	2.63	0.554	1.46	0.77	2.75	0.243	1.19	0.66	2.14	0.569
2	3.58	1.88	6.81	**<0.001**	2.93	1.65	5.22	**<0.001**	2.38	1.39	4.07	**0.002**	1.94	1.19	3.17	**0.008**
3	8.44	4.51	15.79	**<0.001**	6.62	3.76	11.67	**<0.001**	5.44	3.21	9.22	**<0.001**	4.69	2.91	7.57	**<0.001**

Model performances of MELD-Age and ACLF-Age to predict survival at the various time points of 30, 60, 90, and 180 days. Significant values (*p* < 0.05) are highlighted in bold.

Abbreviations: ACLF, acute-on-chronic liver failure; aHR, adjusted hazard ratio; MELD, Model for End-Stage Liver Disease.

## DISCUSSION

Our study demonstrates that patients with severe AH not responding to medical therapy and declined for LT have high short-term and lingering long-term mortality due to ALD and very low rates of subsequent LT. We found that 45% of these declined candidates did not survive beyond 6 months, likely due to the high prevalence of ACLF.^10^ AH-ACLF is associated with reduced response to steroids, higher infection risk, and higher 28-day mortality compared to other ACLF subtypes.^7^,^18^,^19^ Survival correlated well with ACLF grades, and we found similar 28-day mortality in ACLF grade ≥2 (61%, 63/104) as in a recent European study of AH-ACLF (35/53, 66%).^7^ On the other hand, short-term mortality without LT was not universal. Even in those with MELD >40 and/or ACLF-3, 9/51 (18%) survived to 6 months, suggesting a small potential for liver stability allowing for alcohol use disorder treatment or even liver recovery that may obviate the need for LT. However, only 1 patient declined for psychosocial reasons eventually rehabilitated adequately to undergo subsequent transplant. More than one-third of declined patients with available outpatient follow-up data had alcohol relapse, as another recent study demonstrated,^10^ highlighting the need for postdischarge addiction care and the impact of relapse on outcomes (and prediction models) several months after AH diagnosis.^20^

Our results support the growing interest in applying ACLF classification to AH for enhanced prediction and management due to the possible misclassification of mortality by MELD.^21^,^22^ For example, 18% of patients declined for “clinical stability” died within 6 months, suggesting a need for improved prediction models applied at the time of LT waitlist decision-making. In the STOPAH trial, CLIF-C ACLF had a predictive ability similar to that of traditional AH scores.^18^ However, the majority of our cohort would have been excluded from clinical trials due to disease severity: in STOPAH, only 11.3% had ACLF-2 and 1.5% had ACLF-3.^18^ Using multivariable logistic regression, we validated the predictive ability of CLIF-C ACLF and added age to MELD and ACLF grade to derive new practical prediction models. MELD-Age, ACLF-Age, and CLIF-C ACLF had similar prognostic power (AUROC: ~0.73) and performed better than MELD, Lille, and DF. Waitlisted patients with AH have been shown to have lower waitlist mortality than those with other indications for LT when adjusted for MELD.^1^,^23^ Calculating and discussing these 3 prediction models for candidates with AH at transplant selection meetings may provide more accurate 6-month mortality predictions, assisting with waitlist decision-making. For example, in a candidate with severe AH with a borderline psychosocial profile for LT but with age <45 and MELD<35, our results may shift consensus toward deferring waitlisting to allow a trial of outpatient alcohol rehabilitation. However, since psychosocial assessment often drives LT selection more than the severity of liver disease in ALD,^24^ the impact of these improved scoring systems on the selection process is unknown.

Age is a shared variable between CLIF-C ACLF and MELD-Age, and its added prognostic value is somewhat unique to AH. Although most patients with severe AH have cirrhosis, younger patients are more likely to have earlier stages of liver fibrosis, which increases the probability of liver recovery and survival. This is reflected in the inclusion of age (with a similar positive correlation) in AH models such as the Lille model, Age-Bilirubin-International Normalized Ratio-Creatinine score, and the Glasgow alcoholic hepatitis score, and MELD-based scores such as iMELD and MELD for postoperative mortality.^25^,^26^,^27^,^28^ We found that for every 1-year increase in age, there was an 8% increase in the risk of mortality when combined with the MELD score. CLIF-C ACLF includes both age and white blood cells; given the established impact of inflammation, systemic inflammatory response syndrome, and infection in severe AH,^3^,^29^ it is well suited for prognostication in this population but more complicated to calculate.

We derived our prediction models to be used at the time of candidate selection for LT rather than at admission because of the dynamic clinical course during the first week of hospitalization in severe AH, which influences waitlist decision-making. ACLF grade between the third and seventh day after diagnosis predicts outcomes better than the initial grade, which aligns with our center’s admission-to-decision time frame (5 days, median).^11^,^30^ For practicality, we determined cutoffs for rapid clinical use, ACLF grade ≥2, MELD >35, and age >45 years, which were associated with a 2–3-fold increased risk of death at 90 days. These values are remarkably similar to the thresholds in a recent study that used peak MELD, which is less useful.^10^ Regarding candidate selection for LT for AH, younger candidates often elicit more favorable emotional responses and are given the “benefit of the doubt,” but their young age also confers higher transplant-free survival and a greater risk of relapse after LT for AH.^31^,^32^ In candidates with borderline psychosocial profiles with a MELD between 25 and 35, our refined models may influence the decision to defer a patient under 45 years old for waitlisting given their higher likelihood of survival compared to a patient with the same MELD due to metabolic dysfunction–associated steatotic liver disease.

The strengths of our study include prospective patient identification, the largest published cohort of declined candidates for LT for AH to our knowledge, and long-term follow-up using the NDI database. Overall, our cohort was more diverse than that in prior studies: nearly half were women, with higher racial/ethnic minority representation.^5^,^6^,^7^,^10^ This mirrors recent epidemiologic shifts in ALD, with the COVID-19 pandemic being associated with increased alcohol use in younger patients, women, and racially/ethnically diverse populations.^33^ We expand upon the first published experience of LT for AH outside of Europe by determining the natural history of a cohort with life-threatening ALD not traditionally included in prospective studies.^2^,^25^

The limitations of our study include a lack of liver biopsy for AH diagnosis, but all patients met the NIAAA/AASLD diagnostic criteria.^12^ Alcohol data after discharge were not available for about two-thirds of those surviving the index hospitalization. Other potentially significant variables, such as malnutrition, sarcopenia, and frailty,^34^ were not assessed because of the retrospective data analysis. This was a single-center study, which may limit generalizability and highlights the need for future validation of MELD-Age and ACLF-Age as prediction models in this population.

## CONCLUSIONS

Patients with severe AH declined for LT have a high short-term mortality from ACLF and rare subsequent LT. Age added to MELD or ACLF scores enhances survival prediction at the time of waitlist decision in patients with severe AH declined for LT compared to other prediction models. MELD >35, age >45, and ACLF grade ≥2 at the time of LT waitlist decision are practical cutoffs that predict short-term mortality.

## Supplementary Material

SUPPLEMENTARY MATERIAL
