# Supplementary material for: Age added to MELD or ACLF predicts survival in patients with alcohol-associated hepatitis declined for liver transplantation
Source: Hepatol Commun. 2024 Aug 19;8(9):e0514. doi: 10.1097/HC9.0000000000000514 (PMC11340926; doi:10.1097/HC9.0000000000000514)
Supplement: SUPPLEMENTARY MATERIAL [file hc9-8-e0514-s001.docx]

**Supplementary Data:**

**Supplementary Table 1.** Univariable analysis for mortality after waitlist decision.

| **Variable** | **OR** | **95% CI** | ***p* value** |
| --- | --- | --- | --- |
| Age | 1.06 | 1.03 – 1.08 | <0.001 |
| Female sex |  |  | 0.18 |
| Race |  |  |  |
| White |  |  | 0.88 |
| Black |  |  | 0.71 |
| Hispanic/Latinx |  |  | 0.75 |
| Asian |  |  | 0.21 |
| Other |  |  | 0.73 |
| Unknown |  |  | 1.00 |
| Steroid exposure |  |  | 0.75 |
| INR | 1.05 | 1.03 – 1.08 | <0.001 |
| WBC |  |  | 0.19 |
| Bilirubin |  |  | 0.61 |
| Bilirubin trajectory |  |  |  |
| ‘Fast-faller’ |  |  | 0.45 |
| ‘Static’ |  |  | 0.22 |
| ‘Rapid riser’ |  |  | 0.83 |
| Creatinine | 1.45 | 1.17 – 1.79 | 0.001 |
| Need for RRT | 1.95 | 1.003 – 3.77 | 0.049 |
| Sodium |  |  | 0.73 |
| Albumin | 1.57 | 1.10 – 2.24 | 0.01 |
| MAP |  |  | 0.06 |
| SpO2/FiO2 | 0.99 | 0.98 – 1.00 | 0.03 |
| MELD | 1.09 | 1.05 – 1.13 | <0.001 |
| MELD-Na | 1.09 | 1.05 – 1.14 | <0.001 |
| Maddrey’s DF | 1.01 | 1.00 – 1.02 | 0.04 |
| CLIF-C ACLF | 1.11 | 1.06 – 1.15 | <0.001 |
| CLIF-OF | 1.46 | 1.24 – 1.71 | <0.001 |
| Lille Score | 3.74 | 1.62 – 8.62 | 0.002 |
| West Haven Grade for HE |  |  |  |
| 0 |  |  | 0.07 |
| 1 |  |  | 0.05 |
| 2 |  |  | 0.05 |
| 3 |  |  | 0.09 |
| 4 |  |  | 0.99 |
| Use of Vasopressors | 8.38 | 1.08 – 64.95 | 0.04 |
| Mechanical Ventilation | 8.46 | 1.09 – 65.55 | 0.04 |
| ACLF grade |  |  |  |
| 0 |  |  | 0.002 |
| 1 | 1.33 | 0.64 – 2.80 | 0.446 |
| 2 | 1.95 | 0.99 – 3.86 | 0.06 |
| 3 | 5.43 | 2.19 – 13.46 | <0.001 |
| Any ACLF criteria met | 2.24 | 1.30 – 3.87 | 0.004 |
| Liver score |  |  |  |
| 1 |  |  | 0.02 |
| 2 |  |  | 0.09 |
| 3 | 13.38 | 1.61 – 111.01 | 0.02 |
| Liver Dysfunction | 2.68 | 1.30 – 5.52 | 0.01 |
| Kidney score |  |  |  |
| 1 |  |  | 0.01 |
| 2 | 2.72 | 1.09 – 6.78 | 0.03 |
| 3 | 2.35 | 1.25 – 4.45 | 0.01 |
| Kidney Dysfunction | 2.01 | 1.08 – 3.75 | 0.03 |
| Brain score |  |  |  |
| 1 |  |  | 0.002 |
| 2 | 1.97 | 1.09 – 3.58 | 0.03 |
| 3 | 9.78 | 2.21 – 43.22 | 0.003 |
| Brain Dysfunction | 7.78 | 1.78 – 33.95 | 0.01 |
| Coagulation score |  |  |  |
| 1 |  |  | 0.08 |
| 2 |  |  | 0.15 |
| 3 | 2.04 | 1.08 – 3.86 | 0.03 |
| Coagulation Dysfunction |  |  | 0.09 |
| Circulatory score |  |  |  |
| 1 |  |  | 0.01 |
| 2 | 2.81 | 1.09 – 7.25 | 0.03 |
| 3 | 12.26 | 1.59 – 94.36 | 0.02 |
| Circulatory Dysfunction | 10.92 | 1.42 – 83.81 | 0.02 |
| Lung score |  |  |  |
| 1 |  |  | 0.05 |
| 2 |  |  | 1.00 |
| 3 | 12.67 | 1.66 – 96.68 | 0.01 |
| Lung Dysfunction | 12.47 | 1.64 – 95.15 | 0.02 |
| Total Organ dysfunction |  |  | 0.02 |
| 1 |  |  | 0.45 |
| 2 |  |  | 0.07 |
| 3 | 9.00 | 2.05 – 39.50 | 0.004 |
| 4 |  |  | 1.00 |
| 5 | 10.50 | 1.02 – 108.58 | 0.049 |
| 6 |  |  | 1.00 |

**Legend:** Univariable analysis of clinical and laboratory variables at the time of waitlist decision for mortality.

**Abbreviations:**

ACLF: acute-on-chronic liver failure

CLIF-C ACLF: Chronic Liver Failure-Consortium Acute-on-Chronic Liver Failure score

CLIF-OF: Chronic Liver Failure-Consortium Organ Failure score

FiO2: fraction of inspired oxygen

HE: hepatic encephalopathy

INR: international normalized ratio

Maddrey’s DF: Maddrey’s discriminant function

MAP: Mean arterial pressure

MELD: Model for End-Stage Liver Disease

Na: sodium

OR: odds ratio

RRT: renal replacement therapy

SpO2: oxygen saturation

WBC: white blood cell count

**Supplementary Table 2.** Multivariable logistic regression modeling for predictors of mortality after waitlist decision.

| **Baseline Models (bivariate results):** | ***p* value** | | **OR** | **95% CI** |
| --- | --- | --- | --- | --- |
| **ACLF grade** | <0.001 | |  |  |
| 1 | 0.45 | | 1.33 | 0.64 – 2.80 |
| 2 | 0.05 | | 1.95 | 0.99 – 3.86 |
| 3 | <0.001 | | 5.43 | 2.19 – 13.46 |
| **ACLF grade 2 or 3** | <0.001 | | 2.60 | 1.49 – 4.54 |
| **MELD** | <0.001 | | 1.09 | 1.04 – 1.13 |
| **Lille Score** | <0.001 | | 3.73 | 1.62 – 8.62 |
| **CLIF-C ACLF** | <0.001 | | 1.14 | 1.09 – 1.21 |
| **MELD-Age** |  | |  |  |
| Age | <0.001 | | 1.08 | 1.04 – 1.11 |
| MELD | <0.001 | | 1.09 | 1.04 – 1.14 |
| **ACLF-Age** |  | |  |  |
| Age | <0.001 | | 1.05 | 1.03 – 1.08 |
| ACLF-0 | 0.004 | |  |  |
| ACLF-1 | 0.37 | | 1.42 | 0.66 – 3.05 |
| ACLF-2 | 0.09 | | 1.84 | 0.91 – 3.74 |
| ACLF-3 | <0.001 | | 5.45 | 2.14 – 13.86 |
| **1** Age  ACLF grade 2 or 3 | <0.001 | 1.07 | | 1.04 – 1.11 |
|  | 0.03 | 2.20 | | 1.08 – 4.49 |
| **2** Age >45 years  ACLF grade 2 or 3 | <0.001 | 2.66 | | 1.52 – 4.65 |
|  | 0.001 | 2.57 | | 1.45 – 4.55 |
| **3** SpO2/FiO2  Lille Score | 0.04 | 0.99 | | 0.98 – 1.00 |
|  | <0.001 | 5.34 | | 1.99 – 14.33 |
| **4** Age >45 years  MELD | <0.001 | 2.91 | | 1.64 – 5.17 |
|  | <0.001 | 1.09 | | 1.05 – 1.14 |

**Abbreviations:**

ACLF: acute-on-chronic liver failure

CI: confidence interval

CLIF-C ACLF: Chronic Liver Failure-Consortium Acute-on-Chronic Liver Failure score

FiO2: fraction of inspired oxygen

MELD: Model for End-Stage Liver Disease

OR: odds ratio

SpO2: oxygen saturation

**Supplementary Table 3.** Area under the receiver operating characteristic survival curve values (2a) and comparisons between the tested prediction models (2b).

| **Model** | **AUROC** |
| --- | --- |
| ACLF-Age predicted probability | 0.72 |
| CLIF-C ACLF score predicted probability | 0.73 |
| Lille model predicted probability | 0.63 |
| Maddrey’s DF predicted probability | 0.62 |
| MELD-Age predicted probability | 0.73 |

| **Scores compared** | **AUROC Difference** | **95% CI** | ***p* value** |
| --- | --- | --- | --- |
| CLIF-C ACLF vs Lille | **0.10** | **(0.01, 0.18)** | **0.03** |
| CLIF-C ACLF vs ACLF-Age | 0.01 | (-0.04, 0.06) | 0.76 |
| CLIF-C ACLF vs MELD-Age | 0.00 | (-0.05, 0.05) | 0.96 |
| CLIF-C ACLF vs Maddrey’s DF | **0.11** | **(0.02, 0.2)** | **0.02** |
| Lille vs ACLF-Age | **-0.09** | **(-0.17, -0.01)** | **0.03** |
| Lille vs MELD-Age | **-0.10** | **(-0.17, -0.02)** | **0.01** |
| Lille vs Maddrey’s DF | 0.02 | (-0.07, 0.1) | 0.74 |
| ACLF-Age vs MELD-Age | -0.01 | (-0.05, 0.03) | 0.75 |
| ACLF-Age vs Maddrey’s DF | **0.10** | **(0.01, 0.2)** | **0.04** |
| MELD-Age vs Maddrey’s DF | **0.11** | **(0.03, 0.19)** | **0.01** |

**Abbreviations:**

AUROC: area under the receiver operating characteristic curve

CI: confidence interval

CLIF-C ACLF: Chronic Liver Failure-Consortium Acute-on-Chronic Liver Failure score

Maddrey’s DF: Maddrey’s discriminant function

MELD: Model for End-Stage Liver Disease

**Supplementary Table 4.** Analysis of death at 90 days using various CLIF-C ACLF organ score sub-components.

|  |  |  |  |  |  |  |  |  |  |
| --- | --- | --- | --- | --- | --- | --- | --- | --- | --- |
| **Variable** | | **B** | **SE** | **Wald** | **df** | **Exp(B)** | **95.0% CI for Exp(B)** | |  |
|  |  |  |  |  |  |  | **Lower** | **Upper** | ***p* value** |
| Liver Dysfunction | | 1.40 | 0.46 | 9.16 | 1.00 | 4.05 | 1.64 | 10.03 | 0.002 |
| Brain Dysfunction | | 1.17 | 0.52 | 4.98 | 1.00 | 3.22 | 1.15 | 8.96 | 0.03 |
| Coagulation Dysfunction | | 0.77 | 0.22 | 12.26 | 1.00 | 2.16 | 1.40 | 3.32 | <0.001 |
| Kidney Dysfunction | | 0.85 | 0.23 | 13.57 | 1.00 | 2.35 | 1.49 | 3.69 | <0.001 |

|  |  |  |  |  |  |  |  |  |  |
| --- | --- | --- | --- | --- | --- | --- | --- | --- | --- |
| **Variable** | | **B** | **SE** | **Wald** | **df** | **Exp(B)** | **95.0% CI for Exp(B)** | |  |
|  |  |  |  |  |  |  | **Lower** | **Upper** | ***p* value** |
| Liver Dysfunction | | 1.40 | 0.46 | 9.20 | 1.00 | 4.07 | 1.64 | 10.06 | 0.002 |
| Coagulation Dysfunction | | 0.68 | 0.23 | 9.18 | 1.00 | 1.98 | 1.27 | 3.07 | 0.003 |
| Kidney Dysfunction | | 0.83 | 0.24 | 12.43 | 1.00 | 2.29 | 1.45 | 3.63 | <0.001 |

**Legend:** Analysis of subset of patients without lung or circulatory dysfunction to assess death at 90 days by other CLIF-C ACLF organ score sub-components (3a). After removing the variable “brain dysfunction”, other factors’ hazard remained relatively similar (3b).

**Abbreviations:**

B: beta-coefficient

CI: confidence interval

CLIF-C ACLF: Chronic Liver Failure-Consortium Acute-on-Chronic Liver Failure score

df: degrees of freedom

SE: standard error

**Supplementary Table 5**. Proportion of patients surviving at 30, 60, 90 and 180 days, and median time from decision regarding liver transplantation to death according to ACLF grade, MELD score and age. All significant at *p*<0.001.

| **Variable** |  | **Cumulative proportion surviving (days)** | | | | **Median time to death (days)** |
| --- | --- | --- | --- | --- | --- | --- |
|  |  | **30** | **60** | **90** | **180** |  |
| **ACLF Grade 2/3** | No (n=130) | 77% | 69% | 64% | 38% | 700.0 |
|  | Yes (n=104) | 38% | 38% | 35% | 21% | 25.6 |
| **MELD >35** | No (n=138) | 79% | 72% | 68% | 39% | 849.0 |
|  | Yes (n=96) | 32% | 31% | 27% | 19% | 24.0 |
| **Age >45** | No (n=112) | 66% | 61% | 59% | 44% | 702.5 |
|  | Yes (n=122) | 54% | 50% | 44% | 20% | 90.0 |

**Legend:** Proportion of patients surviving at 30, 60, 90 and 180 days, and median time from decision regarding liver transplantation to death according to ACLF grade, MELD score and age. All significant at *p*<0.001.

**Abbreviations:**

ACLF: acute-on-chronic liver failure

MELD: Model for End-Stage Liver Disease
